# Risk of gestational diabetes by aerobic and resistance exercise during the first and second trimesters

**DOI:** 10.1002/pmf2.70246

**Published:** 2026-02-11

**Authors:** Takaki Tanamoto, George Saade, Leryn J. Reynolds, Tetsuya Kawakita

**Affiliations:** ^1^ Department of Obstetrics and Gynecology Teine Keijinkai Hospital Hokkaido Japan; ^2^ Department of Obstetrics and Gynecology Macon & Joan Brock Virginia Health Sciences Eastern Virginia Medical School at Old Dominion University Norfolk Virginia USA; ^3^ Wellness Institute and Research Center, Ellmer College of Health Sciences Macon & Joan Brock Virginia Health Sciences at Old Dominion University Norfolk Virginia USA; ^4^ Center for Maternal and Child Health Equity and Advocacy Eastern Virginia Medical School Norfolk Virginia USA

**Keywords:** aerobic exercise, gestational diabetes mellitus, insufficient exercise, pregnant, resistance exercise

## Abstract

**Objective:**

While some studies have shown that exercise reduces the incidence of gestational diabetes mellitus (GDM), there is limited evidence on whether aerobic or resistance exercise is more beneficial. This study aimed to examine the rate of GDM according to the type of exercise during early pregnancy.

**Study design:**

We analyzed data from the Nulliparous Pregnancy Outcomes Study: Monitoring Mothers‐to‐Be (nuMoM2b), a prospective cohort of individuals enrolled during the first and second trimesters. We excluded participants with pregestational diabetes or delivery <20 weeks. The primary exposure was either aerobic or resistance exercises in the first or second trimester. For each trimester, the metabolic equivalent of task hours per week (MET‐h/week) was calculated for each exercise. Participants were grouped as follows: “Aerobic exercise” (≥7.5 MET‐h/week from aerobic exercises), “Resistance exercise” (≥2 times/week from resistance exercises), “Both types of exercise” (≥7.5 MET‐h/week from aerobic exercise and ≥2 times/week from resistance exercises), and “Insufficient exercise” (≤7.5 MET‐h/week from aerobic exercise and ≤2 times/week from resistance exercises). Our primary outcome was GDM. Adjusted relative risks (aRRs) with 95% confidence intervals (95% CIs) were calculated using modified Poisson regression, adjusting for potential confounders.

**Results:**

In both the first and second trimesters, analyses included 9049 individuals. In the first trimester, individuals who performed aerobic exercise (aRR, 0.75; 95% CI, 0.60–0.93) had a lower risk of GDM compared with those with insufficient exercise. In the second trimester, those who performed aerobic exercise (aRR, 0.78; 95% CI, 0.63–0.97) and both types of exercise (aRR, 0.48; 95% CI, 0.25–0.93) had a lower risk of GDM. Resistance exercise alone, compared to insufficient exercise and compared to aerobic exercise alone, was not clearly associated with significant differences in GDM. Across trimesters, we did not observe increased risks of small for gestational age birth, preterm birth, and preeclampsia.

**Conclusion:**

Aerobic exercise during the first and second trimesters and combined aerobic/resistance exercise during the second trimester were associated with a lower risk of GDM compared to insufficient exercise. Future intervention studies focusing on exercise to reduce the risk of GDM are warranted.

## 1 INTRODUCTION

During pregnancy, exercise benefits both mother and fetus and is recommended by professional organizations such as the World Health Organization (WHO) and the American College of Obstetricians and Gynecologists (ACOG) [1, 2]. According to the WHO, at least 150 min of moderate‐intensity aerobic physical activity per week provides substantial health benefits [1]. ACOG similarly recommends that healthy pregnant women engage in both aerobic and resistance exercises (strength‐conditioning exercises) before, during, and after pregnancy. For resistance exercise specifically, ACOG recommends activity on 2 or more days per week for otherwise healthy pregnant individuals [1]. Although some research shows that prenatal exercise offers cardiovascular and pregnancy benefits for both mother and fetus, the impact of aerobic and resistance exercise on the risk of gestational diabetes (GDM) remains uncertain.

Aerobic and resistance exercises differ in physiological mechanisms and potential effects on pregnancy outcomes. Aerobic exercise (e.g., brisk walking, cycling, running) involves sustained, oxygen‐dependent activity that enhances cardiovascular function [3] and improves insulin sensitivity [4]. Resistance exercise (e.g., pushups, pulling, squatting) engages short‐duration, high‐intensity muscular contractions that increase lean mass, strengthen skeletal muscle, and improve glucose uptake and lipid metabolism [5].

GDM is one of the most common pregnancy complications and is associated with adverse maternal outcomes such as hypertension, preeclampsia, preterm delivery, and hyperinsulinemia [6]. Given these risks, identifying effective, modifiable strategies to reduce GDM incidence is a public health priority. While prior studies suggest that exercise lowers GDM risk overall, the relative contributions of aerobic and resistance exercises have not been thoroughly delineated. The objective of this study was to evaluate the incidence of GDM according to aerobic and resistance exercise participation during pregnancy, with the goal of clarifying whether these exercise modalities differ in their associations with GDM risk.

## 2 MATERIALS AND METHODS

This study was a secondary analysis of prospectively collected data from the Nulliparous Pregnancy Outcomes study: Monitoring Mothers‐to‐Be (nuMoM2b), conducted between 2010 and 2014. A total of 10,038 participants were enrolled across eight US academic centers (Case Western Reserve University, Cleveland, Ohio; Columbia University, New York, New York; Indiana University, Indianapolis, Indiana; University of Pittsburgh, Pittsburgh, Pennsylvania; Northwestern University, Chicago, Illinois; University of California at Irvine, Irvine, California; University of Pennsylvania, Philadelphia, Pennsylvania; and University of Utah, Salt Lake City, Utah) [7]. Each site obtained Institutional Review Board (IRB) approval, and all participants provided written informed consent at enrollment. Individuals with pregestational diabetes, those who delivered before 20 weeks of gestation, or those with missing exercise data during the first and second trimester were excluded. This secondary analysis followed the Strengthening the Reporting of Observational Studies in Epidemiology (STROBE) guidelines for cohort studies. Because the analytic dataset was fully de‐identified, our institution determined that the project met the criteria for IRB exemption.

Nulliparous individuals were enrolled between 6 0/7 weeks’ gestation and 13 6/7 weeks of gestation. The original nuMom2b cohort excluded individuals younger than 13 years, those with more than or equal to three prior spontaneous abortions, known major fetal anomalies or aneuploidy at enrollment, assisted reproduction with donor oocytes, multifetal reduction, and participation in any interventional study that could alter maternal or fetal outcomes. Trained study staff administered interviews at three time points (6–13 weeks’ gestation [visit 1], 16–21 weeks’ gestation [visit 2], and 22–29 weeks’ gestation [visit 3]) using standardized physical activity questions adapted from the Behavioral Risk Factor Surveillance System (BRFSS). These interviews captured details on the type, duration, and frequency of structured exercise. For each participant, up to three distinct exercise modalities were recorded, including duration (minutes per session), frequency (sessions per week), and, when applicable, distance covered. Trained coders used the Physical Activity Compendium to determine the metabolic equivalent of task (MET) for each reported activity, thereby obtaining a single weekly exercise‐intensity metric for categorization (MET‐h/week) [8]. These MET‐h/week values reflected only leisure‐time, structured exercise, and did not include occupational or household activity.

### 2.1 Exposure

Exercise information from visit 1 (first trimester) or visit 2 (early second trimester) was available for 9049 individuals. Although exercise data were collected at three visits, we restricted the analysis to visits 1 and 2 to avoid potential bias after GDM diagnosis, which may have occurred before visit 3.

Reported physical activity types were classified as aerobic or resistance and are summarized in Table 1 [5]. For each participant, we summed the total MET‐h/week separately for aerobic activities reported at each visit.

**TABLE 1:** Categorization of physical activities by exercise type.

	Type of exercises	
	Aerobic class/exercise machines	Running
Aerobic exercises	Basketball	Skating—Ice or roller
	Bowling	Sledding/Tobogganing
	Canoeing/rowing/sailing	Snow blowing
	Cycling	Softball/Baseball
	Dancing	Stair climbing
	Fishing	Swimming
	Gardening	Table tennis
	Golf	Tennis
	Handball/squash	Volleyball
	Hiking/backpacking	Walking
	Jogging	Yoga
	Judo/karate/tae kwon do	
**Resistance exercises**	Calisthenics/home or gym exercise	Weightlifting

*Note*: Classification was based on the American College of Sports Medicine (ACSM) definitions: Aerobic activities involve large muscle groups with reliance on oxygen metabolism. Resistance activities are short‐duration, high‐intensity efforts typically relying on anaerobic pathways.

Participants were categorized into four groups based on their cumulative weekly exercise intensity: “Aerobic exercise” (≥7.5 MET‐h/week from aerobic exercises), “Resistance exercise” (≥2 times/week from resistance exercises), “Both types of exercise” (≥7.5 MET‐h/week from aerobic exercise and ≥2 times/week from resistance exercises), and “Insufficient exercise” (≤7.5 MET‐h/week from aerobic exercise and ≤2 times/week from resistance exercises). We selected 7.5 METs‐h/week as the primary cutoff for aerobic activity because it corresponds to the minimum weekly volume of moderate‐intensity activity recommended by ACOG (at least 150 min of moderate‐intensity aerobic activity per week, where moderate intensity is defined as 3.0–5.9 METs, equivalent to approximately 7.5–14.9 MET‐h/week) [1]. In contrast, resistance exercise was evaluated using frequency rather than METs. Unlike aerobic exercise, resistance exercise primarily involves muscle contraction and mechanical load rather than sustained oxygen consumption. Therefore, MET‐based quantification may not accurately capture its definition. The frequency of exercise is widely used and considered a more appropriate indicator of resistance exercise [9]. The threshold of ≥2 resistance exercise sessions per week was chosen based on Australian prenatal exercise guidelines, which recommend resistance exercise on at least 2 days/week to support maternal health [10].

### 2.2 Outcomes

Our primary outcome was GDM. We also examined secondary outcomes, including small for gestational age (SGA) birth (<10th percentile), preterm labor (<37 weeks’ gestation), and preeclampsia. Detailed definitions of outcomes are presented in Method S1.

### 2.3 Statistical analysis

Descriptive statistics were generated to summarize participant characteristics by exercise‐type group (Aerobic, Resistance, Both, and Insufficient exercise) and trimester. Categorical variables were summarized as counts and percentages, and continuous variables as means with standard deviations. Maternal age and body mass index (BMI) were treated as continuous variables, while all other covariates were included as categorical variables using dummy coding. Race and ethnicity were combined into a single mutually exclusive variable as defined in the nuMoM2b dataset. While we recognize that race is a social construct and not a biological factor, it was included as a covariate due to its known association with GDM risk, particularly among Asian and Black/African American individuals [11].

Primary analyses were conducted separately for the first and early second trimesters to examine trimester‐specific associations between exercise type and GDM. Adjusted risk ratios (aRRs) with 95% confidence intervals (95% CIs) were calculated using modified Poisson regression with robust error variance, adjusting for potential confounders, including maternal age, BMI, smoking status, race, family income, energy intake, and conception via assisted reproductive technology (ART) [12, 13]. We assessed multicollinearity between variables using generalized variance inflation factors (GVIFs). All adjusted GVIF values were close to 1.0, indicating no meaningful multicollinearity among covariates (Table S1).

Each model included one observation per participant and, therefore, random effects for within‐subject correlation were not required. “Insufficient exercise” served as the reference group. This approach was also used to analyze secondary outcomes, including SGA birth, preterm birth, and preeclampsia. We also reported aRRs with 95% CIs comparing resistance exercise and combined exercise with aerobic exercise alone.

### 2.4 Missing information

Information on missing variables is summarized in Table S2. Because some variables had >5% missingness [14], we conducted multiple imputation using chained equations (MICE). The imputation model included all outcomes and key covariates, including maternal age, BMI, smoking status, race, family income, energy intake, ART, and exercise type (exposures). Binary outcomes were imputed using logistic regression, continuous variables using predictive mean matching, and categorical variables using multinomial logistic regression. All variables were permitted to serve as predictors. Twenty imputed datasets were generated with 20 iterations each, and subsequent analyses were pooled using Rubin's rules [15].

### 2.5 Sensitivity analysis

We conducted several sensitivity analyses to assess the robustness of our findings. First, we repeated the primary analysis using a complete‐case approach, restricting the sample to participants with no missing data on the outcome, exposure, or covariate. Second, to evaluate whether lower levels of physical activity were associated with GDM, we redefined exercise exposure using a lower aerobic threshold of 5 MET‐h/week instead of 7.5 MET‐h/week, corresponding to approximately 60 min of brisk walking per week [8], to assess whether minimal activity levels influenced the associations. We also redefined resistance exercise as ≥1 resistance exercise session per week. The analyses were repeated using the same modified Poisson regression models and covariate adjustments as in the primary analysis. Third, to evaluate the linearity of the association between aerobic exercise intensity and the risk of GDM, we fit modified Poisson regression models with robust variance, incorporating restricted cubic splines for aerobic METs to allow for nonlinearity. The model was adjusted for age, race, BMI, family income, infertility treatment, smoking, and resistance exercise (≥2 times a week vs. <2 times a week). Competing models with three and four knots were compared using Akaike and Bayesian Information Criteria, and the three‐knot model demonstrated superior fit; thus, it was selected as the final specification. Linearity was assessed by testing the highest‐order spline term. Fourth, when linearity was supported, we estimated the adjusted risk reduction per 5 MET‐h/week as well as with resistance exercise (≥2 times/week vs. <2 times/week). We fit modified Poisson regression models with robust variance to estimate aRRs and 95% CIs, including an interaction term between aerobic and resistance exercises. All models were adjusted for maternal age, BMI, smoking status, race, family income, energy intake, and ART. All statistical analyses were conducted using R version 4.4.2 (R Foundation for Statistical Computing).

## 3 RESULTS

**FIGURE 1:**
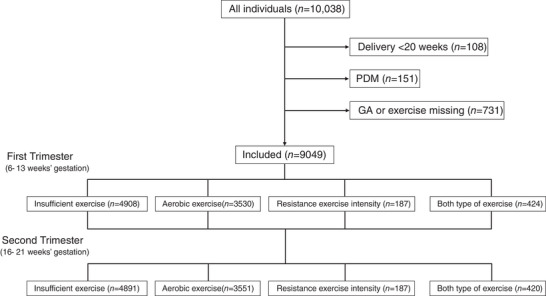
Cohort diagram. GA, gestational age;PDM, pregestational diabetes.

Of the 10,038 individuals initially enrolled, 9049 were included in the analytic cohort (Figure 1). At visit 1, 4908 participants (54.2%) reported insufficient exercise, 3530 (39.0%) engaged in aerobic exercise, 187 (2.1%) engaged in resistance exercise, and 424 (4.7%) engaged in both exercise types. At visit 2, 4891 participants (54.1%) reported insufficient exercise, 3551 (39.2%) engaged in aerobic exercise, 187 (2.1%) engaged in resistance exercise, and 420 (4.6%) engaged in both.

Demographic characteristics are presented in Tables 2 and 3. Significant differences were observed across exercise groups in maternal age, race, smoking status, family income, and chronic hypertension (all *p* < 0.001). ART use also differed across groups at visit 2 (*p* < 0.001), but not at visit 1 (*p* = 0.103).

**TABLE 2:** Demographics according to the exercise type (first trimester).

	Insufficient (*n*=4908)	Aerobic (*n*=3530)	Resistance (*n*=187)	Both (*n*=424)	*p* value
Age (years)	26.1 (5.6)	28.0 (5.5)	28.3 (5.6)	30.1 (4.3)	<0.001
BMI (kg/m^2^)	26.9 (6.7)	25.6 (5.6)	25.8 (5.7)	24.3 (4.0)	<0.001
Aerobic (METs‐h/week)	0.0 [0.0, 3.5]	16.5 [10.9, 25.9]	3.5 [0.0, 5.5]	17.5 [11.8, 27.1]	<0.001
Frequency of resistance exercise per week	0 [0, 0]	0 [0, 0]	3 [2, 4]	3 [2, 3]	<0.001
Energy intake	1542 [1158, 2065]	1507 [1185, 1893]	1446 [1098, 1818]	1550 [1238, 1886]	0.002
Race					<0.001
Asian	183 (3.7)	159 (4.5)	8 (4.3)	20 (4.7)	
Black	819 (16.7)	347 (9.8)	18 (9.6)	13 (3.1)	
Hispanic	991 (20.2)	409 (11.6)	23 (12.3)	31 (7.3)	
White	2678 (54.6)	2412 (68.3)	130 (69.5)	352 (83.0)	
None of above	237 (4.8)	203 (5.8)	8 (4.3)	8 (1.9)	
Smoking	960 (19.6)	534 (15.1)	30 (16.0)	46 (10.8)	<0.001
Artificial reproductive technology	182 (3.7)	155 (4.4)	8 (4.3)	25 (5.9)	0.103
Family income ≥$100,000	1026 (27.1)	1368 (44.5)	88 (53.7)	252 (62.5)	<0.001
Cesarean delivery	1348 (27.7)	932 (26.6)	52 (28.0)	112 (26.7)	0.732
Chronic hypertension	131 (2.7)	57 (1.6)	5 (2.7)	3 (0.7)	0.001

*Note*: Categorical variables are represented as *n* (%). Continuous variables are represented as mean (SD) or median [25th, 75th percentile].

Statistical comparisons were conducted using one‐way ANOVA for age; Kruskal–Wallis test for BMI; and chi‐square tests for categorical variables, including race/ethnicity, marital status, education, insurance type, smoking status, and chronic hypertension.

Abbreviations: BMI, body mass index; GED, general education development; IQR, interquartile range; MET, metabolic equivalent.

**TABLE 3:** Demographics according to the exercise type (Early second trimester).

	Insufficient (*n*=4891)	Aerobic (*n*=3551)	Resistance (*n*=187)	Both (*n*=420)	*p* value
Age (years)	26.0 (5.6)	28.0 (5.5)	28.8 (5.3)	30.4 (4.2)	<0.001
BMI (kg/m^2^)	27.1 (6.7)	25.4 (5.6)	24.77 (5.1)	23.99 (3.8)	<0.001
Aerobic (METs‐h/week)	0.0 [0.0, 4.1]	15.2 [10.5, 23.4]	2.8 [0.0, 5.3]	15.4 [11.0, 21.8]	<0.001
Frequency of resistance exercise per week	0 [0, 0]	0 [0, 0]	3 [2, 4]	2 [2, 3]	<0.001
Energy intake (kcal/day)	1540 [1159, 2049]	1512 [1189, 1917]	1405 [1094, 1843]	1527 [1197, 1859]	0.021
Race					<0.001
Asian	184 (3.8)	156 (4.4)	12 (6.4)	18 (4.3)	
Black	807 (16.5)	359 (10.1)	20 (10.7)	11 (2.6)	
Hispanic	971 (19.9)	437 (12.3)	20 (10.7)	26 (6.2)	
White	2684 (54.9)	2406 (67.8)	130 (69.5)	352 (83.8)	
None of above	245 (5.0)	193 (5.4)	5 (2.7)	13 (3.1)	
Smoking	984 (20.1)	532 (15.0)	16 (8.6)	38 (9.0)	<0.001
Artificial reproductive technology	167 (3.4)	160 (4.5)	10 (5.3)	33 (7.9)	<0.001
Family Income ≥$100,000	1018 (27.1)	1377 (44.3)	78 (48.4)	261 (64.1)	<0.001
Cesarean delivery	1371 (28.2)	906 (25.7)	55 (29.6)	112 (26.8)	0.064
Chronic hypertension	137 (2.8)	54 (1.5)	4 (2.2)	1 (0.2)	<0.001

*Note*: Numbers are shown as *n* (%), mean (SD), and median [25th, 75th percentile]. Statistical comparisons were conducted using one‐way ANOVA for age; Kruskal–Wallis test for BMI; and chi‐square tests for categorical variables, including race/ethnicity, marital status, education, insurance type, smoking status, and chronic hypertension.

Abbreviations: BMI, body mass index; GED, general education development; IQR, interquartile range MET, metabolic equivalent.

**FIGURE 2:**
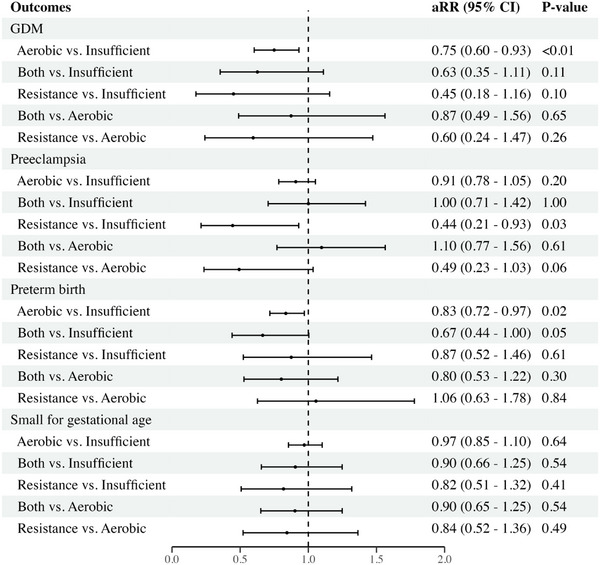
Adjusted relative risks for outcomes by type of exercise in the first trimester. These results are based on modified Poisson regression with robust error variance, adjusting for potential confounders, including maternal age, BMI, smoking status, race, family income, and conception via assisted reproductive technology (ART). Estimates were obtained from multiple imputations. CI, confidence interval; GDM, gestational diabetes; RR, relative risk.

Relative risks according to the type of exercise at visit 1 are presented in Figure 2, with frequencies and proportions provided in Table S3. In the first trimester, compared with the insufficient exercise group, aerobic exercise had a significantly lower risk of GDM (aRR, 0.75; 95% CI, 0.60–0.93). Women who engaged in resistance exercise alone or in combination with aerobic exercise had a lower risk of GDM compared with those who had insufficient exercise, but the difference did not reach statistical significance (aRR, 0.45; 95% CI, 0.18–1.16 and aRR, 0.63; 95% CI, 0.35–1.11, respectively). No significant associations were observed between exercise type and SGA birth, preterm birth, or preeclampsia. Additionally, there was no significant difference between aerobic exercise alone versus resistance exercise alone in the rates of any of the outcomes. Furthermore, resistance exercise alone and combined exercise did not differ significantly from aerobic exercise alone with respect to GDM or other adverse outcomes.

**FIGURE 3:**
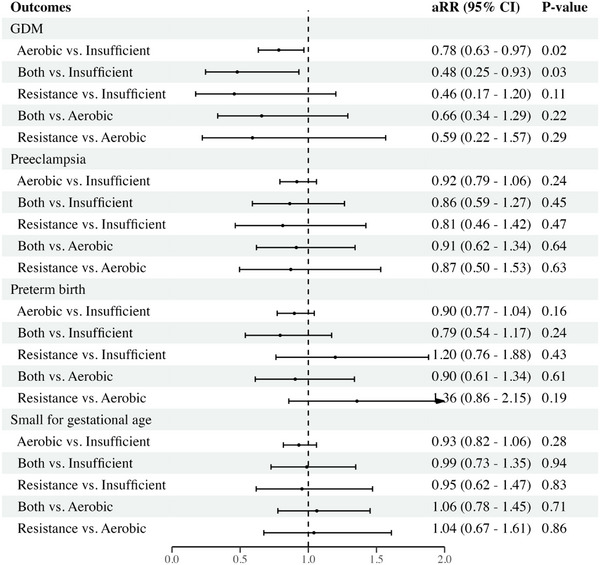
Adjusted relative risks for outcomes by type of exercise in the early second trimester. These results are based on modified Poisson regression with robust error variance, adjusting for potential confounders, including maternal age, BMI, smoking status, race, family income, and conception via assisted reproductive technology (ART). Estimates were obtained from multiple imputations. CI, confidence interval; GDM, gestational diabetes mellitus; RR, Relative Risk.

Relative risks according to the type of exercise at visit 2 are presented in Figure 3, with frequencies and proportions provided in Table S4. In the early second trimester, compared to insufficient exercise, aerobic exercise (aRR, 0.78; 95% CI, 0.63–0.97) and a combination of aerobic and resistance exercises (aRR, 0.48; 95% CI, 0.25–0.93) were associated with lower GDM risk, while resistance exercise alone showed no significant association (aRR, 0.46; 95% CI, 0.17–1.20). No significant associations were observed between exercise type and SGA birth, preterm birth, or preeclampsia. Resistance exercise alone and combined exercise also did not differ significantly from aerobic exercise alone for any of the outcomes.

We conducted several sensitivity analyses to assess the robustness of our study. First, we repeated the primary analysis using a complete‐case approach (Figure S1). Results were similar to the primary analysis, suggesting that missing data did not influence the findings. Second, we redefined the aerobic exercise exposure using a lower threshold of 5 MET‐h/week instead of 7.5 MET‐h/week and resistance exercise as ≧1 session per week. Overall, the direction of the associations between exercise type and GDM remained consistent with the primary analysis (Figure S2). In the first trimester, compared to insufficient exercise, aerobic exercise alone and in combination with resistance exercise were associated with a decreased risk of GDM (aRR, 0.74; 95% CI, 0.60–0.91 and aRR, 0.54; 95% CI, 0.33–0.88, respectively). In the early second trimester, compared to insufficient exercise, aerobic exercise alone, in combination with resistance exercise, and resistance exercise alone were associated with a decreased risk of GDM (aRR, 0.71; 95% CI, 0.58–0.88; aRR, 0.48; 95% CI, 0.28–0.81; and aRR, 0.31; 95% CI, 0.10–0.96, respectively). In this sensitivity analysis, compared to insufficient exercise, aerobic exercise was also associated with a decreased risk of preeclampsia (aRR, 0.85; 95% CI, 0.73–0.98) during the first trimester and preterm birth (aRR, 0.82; 95% CI, 0.71–0.96) during the early second trimester.

Third, in both the first and early second trimesters, the spline terms were not statistically significant (*p* = 0.34 and *p* = 0.12, respectively), supporting a linear association between aerobic exercise intensity and GDM risk. Fourth, in models including an interaction term between aerobic exercise (measured in MET‐h/week) and binary resistance exercise, each additional 5 MET‐h/week of aerobic activity was associated with a 7% reduction in the risk of GDM in the first trimester (aRR 0.93; 95% CI, 0.88–0.98) and a 9% reduction in the early second trimester (aRR 0.91; 95% CI, 0.86–0.96). Resistance exercise (≥2 times/week vs. <2 times/week) was also associated with a lower risk of GDM in both trimesters (first trimester: aRR, 0.48; 95% CI, 0.24–0.95; early second trimester: aRR, 0.44; 95% CI, 0.21–0.92). Interaction *p* values were not significant (*p* = 0.10 for the first trimester and *p* = 0.30 for the second trimester).

## 4 DISCUSSION

### 4.1 Principal findings

In this secondary analysis of the prospective nuMoM2b cohort, engagement in aerobic exercise during both the first and second trimesters was associated with a reduced risk of GDM. Additionally, combined aerobic and resistance exercise in the second trimester was associated with a lower risk of GDM, whereas resistance exercise alone was inconclusive and should be interpreted cautiously, given limited statistical power. Notably, when modeled continuously, each 5 MET‐h/week increase in aerobic activity was associated with a 7%–9% lower GDM risk across trimesters, and resistance exercise remained beneficial. Importantly, across trimesters and exercise types, we did not observe increased risks of SGA, preterm birth, and preeclampsia.

### 4.2 Results in the context of what is known

Although aerobic and resistance exercises are both recognized as beneficial components of physical activity, prior research has largely emphasized overall activity volume or moderate–vigorous intensity rather than distinguishing specific exercise modalities [16]. Notably, the randomized controlled trial (RCT) [17] and prospective RCT [18] study evaluating prenatal exercise interventions have primarily compared intervention groups with usual care or control groups, without directly comparing aerobic and resistance exercise modalities. In contrast, our study directly compared aerobic exercise (either alone or in combination with resistance exercise), resistance exercise alone, and insufficient exercise, allowing a more detailed evaluation of how specific exercise modalities may differentially relate to reducing GDM risk.

### 4.3 Clinical implications

Our findings indicate that both the timing and modality of exercise are relevant for reducing the risk of GDM. Aerobic activity in the first and early second trimesters—and combined aerobic and resistance exercise in the early second trimester—was associated with a lower risk of GDM, even after adjusting for confounders and testing for linearity. Resistance exercise alone did not show a clear benefit, although small subgroup sizes limit definitive conclusions. These findings support counseling pregnant individuals to engage in routine aerobic activity, with or without simple resistance exercise, tailored to fitness level and pregnancy course.

Several physiologic pathways may account for these associations. Aerobic exercise improves insulin sensitivity and promotes skeletal–muscle glucose uptake [19], while resistance exercise primarily increases or preserves muscle mass, expanding long‐term capacity for glucose disposal [20].

Clinically, prenatal counseling may benefit from moving beyond general advice to “remain active” toward emphasizing routine aerobic activity supplemented by simple, pregnancy‐appropriate resistance exercises tailored to individual fitness and obstetric risk.

### 4.4 Research implications

Future studies should clarify the physiological mechanisms through which aerobic and resistance exercises, individually and in combination, affect glucose metabolism during pregnancy. Detailed assessments of insulin sensitivity, substrate utilization, and pregnancy‐specific metabolic adaptations are needed to elucidate how exercise reduces GDM risk. Long‐term follow‐up of mothers and offspring will help determine whether prenatal exercise patterns influence postpartum metabolic health, childhood adiposity, or later type 2 diabetes [21].

Because this analysis is observational, the findings should be considered hypothesis‐generating. RCTs that isolate aerobic and resistance components, use structured and standardized exercise regimens, and enroll socioeconomically and racially diverse populations are essential to confirm these associations and strengthen generalizability for future clinical guidelines.

### 4.5 Strengths and limitations

A major strength of this study is its large, multicenter, prospective design with enrollment early in pregnancy and visit‐specific characterization of exercise type and intensity, along with detailed protocol and definitions. The data and outcomes were rigorously collected by trained research staff. In addition, we classified participants into four groups—insufficient exercise, aerobic exercise, resistance exercise, and combined aerobic plus resistance—using clinically meaningful, guideline‐based thresholds (≥7.5 MET‐h/week for aerobic activity and ≥2 resistance sessions/week). This approach allowed us to distinguish real‐world exercise behaviors with greater granularity than studies that categorize physical activity solely by total volume or intensity. Applying this framework separately to the first and second trimesters enabled us to identify that aerobic and combined exercise patterns were most consistently associated with reduced GDM risk, offering clinically relevant insights for prenatal counseling.

The study also has limitations. Exercise was self‐reported, introducing potential recall and social desirability biases. However, prior studies have demonstrated that among pregnant women, self‐reported physical activity measures, including frequency, duration, and intensity, show acceptable validity and reliability [22]. Although the questionnaire distinguished aerobic from resistance modalities, granular details of resistance exercise (such as load, repetitions, sets, and rest intervals) were unavailable, and some participants likely performed multiple strength modalities on the same day. Such misclassification may have attenuated associations involving resistance exercise. The cohort consisted exclusively of nulliparous individuals receiving care at academic centers, which may limit generalizability to multiparous patients or those in community and rural settings, where exercise patterns and access to structured programs differ. Furthermore, real‐world activities often include mixed aerobic and resistance components, and our three‐category classification may not fully capture this complexity.

Finally, the number of both resistance and combined exercise groups was small, which may have limited the statistical power to detect differences. To address this, we additionally modeled aerobic exercise as a continuous variable and resistance exercise as a binary variable (≥2 times/week vs. <2 times/week). In addition, because multiple secondary outcomes were examined, these analyses should be interpreted cautiously as exploratory. These models supported a linear association between aerobic activity and GDM risk and also demonstrated a significant association between resistance exercise and lower GDM risk, despite the smaller subgroup size.

## 5 CONCLUSIONS

In conclusion, this study suggests that aerobic exercise (alone or in combination with resistance exercise) during early and mid‐pregnancy is associated with a lower risk of GDM. While these findings support the potential value of incorporating aerobic activity into prenatal care, they should be interpreted within the limitations of observational data. Further randomized trials are needed to determine whether specific exercise modalities can be recommended for reducing the risk of GDM in clinical practice.

## CONFLICT OF INTEREST STATEMENT

The authors declare no conflicts of interest.

## ETHICS STATEMENT

This study was determined to be exempt from review by the Institutional Review Board (IRB).

## Supporting information



Supporting information

## Data Availability

The data that support the findings of this study are available from the corresponding author upon reasonable request.
