# Supplementary material for: Risk of gestational diabetes by aerobic and resistance exercise during the first and second trimesters
Source: Pregnancy (Hoboken). 2026 Feb 11;2(2):e70246. doi: 10.1002/pmf2.70246 (PMC13344322; doi:10.1002/pmf2.70246)
Supplement: Supplementary file 1 [file PMF2-2-e70246-s001.docx]

**Online-Only Supplements**

Title: Risk of gestational diabetes by aerobic and anaerobic exercise during the first and second trimesters.

Takaki TANAMOTO, MD; Tetsuya KAWAKITA, MD, MS

**Supplemental Method 1.** Definitions of outcomes

**Supplemental Table 1.** Assessment of multicollinearity

**Supplemental Table 2.** Missing information

**Supplemental Table 3.** Frequencies and Proportions of Participants with Adverse Pregnancy Outcomes by Exercise Intensity at Visit 1(First Trimester)

**Supplemental Table 4.** Frequencies and Proportions of Participants with Adverse Pregnancy Outcomes by Exercise Intensity at Visit 2 (Early Second Trimester)

**Supplemental Figure 1.** Adjusted relative risks for outcomes by type of exercise using complete case analysis.

**Supplemental Figure 2.** Adjusted relative risks and 95% confidence intervals for adverse pregnancy outcomes by exercise types, based on sensitivity analyses redefining aerobic exercise exposure using 5 MET-hr/week and resistance exercise as ≧1 session per week.

**Supplemental Method 1.** Definitions of Exposure and Outcomes

Gestational diabetes was defined by one of the following oral glucose tolerance test (OGTT) criteria: 1) 100-gram 3-hour diagnostic OGTT with two or more abnormal values (fasting ≥95 mg/dL, 1-hour ≥180 mg/dL, 2-hour ≥155 mg/dL, or 3-hour ≥140 mg/dL); 2) 75-gram 2-hour diagnostic OGTT with one or more abnormal values (fasting ≥92 mg/dL, 1-hour ≥180 mg/dL, 2-hour ≥153 mg/dL. OGTT was performed from 24 to 28 weeks’ gestation.^[1]^

Preterm birth was defined as delivery before 37 weeks’ gestation, either spontaneous or medically indicated.^[2]^

SGA was defined as a birth weight below the 10th percentile for gestational age, based on Alexander's fetal growth curves.^[3]^

Preeclampsia was defined as new-onset hypertension (systolic blood pressure ≥140 mmHg or diastolic blood pressure ≥90 mmHg) occurring after 20 weeks of gestation, most commonly near term. It is often accompanied by new-onset proteinuria (≥300 mg/day, urine protein/creatinine ratio ≥0.3); however, in some cases, preeclampsia may present without proteinuria but with other clinical signs or symptoms.^[4]^

Pregestational diabetes was defined as a patient who had already been diagnosed with type 1 or type 2 diabetes before the onset of pregnancy.^[5]^

**Supplemental Table 1.** Assessment of multicollinearity

|  | Model 1 (Visit 1) | | | Model 2 (Visit 2) | | |
| --- | --- | --- | --- | --- | --- | --- |
| Variable | GVIF | Df | GVIF^(1/(2*Df)) | GVIF | Df | GVIF^(1/(2*Df)) |
| Exercise type | 1.08 | 3 | 1.01 | 1.08 | 3 | 1.01 |
| Age | 1.23 | 1 | 1.11 | 1.23 | 1 | 1.11 |
| Body mass index | 1.12 | 1 | 1.06 | 1.12 | 1 | 1.06 |
| Smoking | 1.10 | 1 | 1.05 | 1.09 | 1 | 1.05 |
| Race | 1.01 | 1 | 1.01 | 1.01 | 1 | 1.01 |
| Income | 1.27 | 1 | 1.13 | 1.27 | 1 | 1.12 |
| ART | 1.07 | 1 | 1.03 | 1.07 | 1 | 1.03 |
| Energy intake | 1.11 | 1 | 1.06 | 1.11 | 1 | 1.05 |

Abbreviations: ART (artificial reproductive technology), Df (degree of freedom), GVIF (generalized variance inflation factors)

**Supplemental Table 2.** Missing information

|  | Missing (%) |
| --- | --- |
| Age (year) | 0/9049 (0.0) |
| Early pregnancy BMI (kg/m^2^) | 143/9049 (1.6) |
| Aerobic exercise | 0/9049 (0.0) |
| Resistance exercise | 0/9049 (0.0) |
| Energy intake | 1336/9049 (14.8) |
| Race | 0/9049 (0.0) |
| Smoking before pregnancy | 3/9049 (0.0) |
| ART | 2/9049 (0.0) |
| Family income | 1620/9049 (17.9) |
| Chronic hypertension | 67/9049 (0.7) |
| Gestational diabetes | 220/9049 (2.4) |
| Preeclampsia | 0/9049 (0.0) |
| Preterm birth | 70/9049 (0.8) |
| Small for gestational age | 0/9049 (0.0) |
|  |  |

Abbreviations: ART (assisted reproductive technology); BMI (body mass index); IVF (in vitro fertilization)

**Supplemental Table 3.** Number and Percentage of Participants with Adverse Pregnancy Outcomes by Exercise Intensity at Visit 1(First Trimester)

|  | **Insufficient exercise**  **(n=4,908)** | **Aerobic exercise**  **(n=3,530)** | **Resistance exercise**  **(n=187)** | **Both type of exercises**  **(n=424)** |
| --- | --- | --- | --- | --- |
|  | n (%) | n (%) | n (%) | n (%) |
| GDM | 242 (4.9) | 126 (3.6) | 4 (2.1) | 12 (2.8) |
| SGA | 544 (11.1) | 356 (10.1) | 16 (8.6) | 37 (8.7) |
| Preterm birth | 447 (9.1) | 255 (7.2) | 14 (7.5) | 23 (5.4) |
| Preeclampsia | 457 (9.3) | 270 (7.6) | 7 (3.7) | 32 (7.5) |

Abbreviations: GDM (gestational diabetes mellitus); SGA (small for gestational age)

**Supplemental Table 4.** Number and Percentage of Participants with Adverse Pregnancy Outcomes by Exercise Intensity at Visit 2 (Early Second Trimester)

|  | **Insufficient exercise**  **(n=4,891)** | **Aerobic exercise**  **(n=3,551)** | **Resistance exercise**  **(n=187)** | **Both type of exercises**  **(n=420)** |
| --- | --- | --- | --- | --- |
|  | n (%) | n (%) | n (%) | n (%) |
| GDM | 240 (4.9) | 131 (3.7) | 4 (2.1) | 9 (2.1) |
| SGA | 545 (11.1) | 349 (9.8) | 19 (10.2) | 40 (9.5) |
| Preterm birth | 430 (8.8) | 265 (7.5) | 18 (9.6) | 26 (6.2) |
| Preeclampsia | 455 (9.3) | 272 (7.7) | 12 (6.4) | 27 (6.4) |

Abbreviations: GDM (gestational diabetes mellitus); SGA (small for gestational age)

**Supplemental Figure 1.** Adjusted relative risks for outcomes by type of exercise using complete case analysis.

Abbreviations: CI (confidence interval); GDM (gestational diabetes); RR (relative risk)

Adjusted relative risks (aRR) and 95% confidence intervals (CI) are shown for adverse pregnancy outcomes: (a) Visit 1and (b) Visit 2.

These results are based on modified Poisson regression with robust error variance, adjusting for potential confounders, including maternal age, BMI, smoking status, race, family income, energy intake, and conception via assisted reproductive technology.

**Supplemental Figure 2.** Adjusted relative risks and 95% confidence intervals for GDM and other adverse pregnancy outcomes by exercise types, based on sensitivity analyses redefining aerobic exercise exposure using 5 MET-hr/week and resistance exercise as ≧1 session per week.

Adjusted relative risks (aRR) and 95% confidence intervals (CI) are shown for adverse pregnancy outcomes: (a) Visit 1and (b) Visit 2. Estimates were obtained from multiple imputations. Models were adjusted for maternal age, BMI, smoking status, race, family income, energy intake, and conception via assisted reproductive technology.

Reference:

1. “ACOG Practice Bulletin No. 190: Gestational Diabetes Mellitus,” *Obstetrics & Gynecology* 131, no. 2 (2018): e49–64, https://doi.org/10.1097/AOG.0000000000002501.
2. “Practice Bulletin No. 171: Management of Preterm Labor,” *Obstetrics & Gynecology* 128, no. 4 (2016): e155, https://doi.org/10.1097/AOG.0000000000001711.
3. “ACOG Practice Bulletin No. 227: Fetal Growth Restriction,” *Obstetrics & Gynecology* 137, no. 2 (February 2021): e16–28, https://doi.org/10.1097/AOG.0000000000004251.
4. “Gestational Hypertension and Preeclampsia: ACOG Practice Bulletin, Number 222,” *Obstetrics and Gynecology* 135, no. 6 (2020): e237–60, https://doi.org/10.1097/AOG.0000000000003891.
5. Brittany N. Hart et al., “Pregestational Diabetes and Family Planning,” *Clinical Diabetes : A Publication of the American Diabetes Association* 39, no. 3 (2021): 323–28, https://doi.org/10.2337/cd20-0062.
